# Research on the effect of water-cooling steel pipe on preventing spontaneous combustion of coal pile and its thermal migration behavior

**DOI:** 10.1038/s41598-024-58857-3

**Published:** 2024-04-17

**Authors:** Chunming Ai, Shuang Xue, Li Zhang, Qinyuan Zhou

**Affiliations:** 1 College of Safety Science and Engineering, Liaoning Technical University, Huludao, 125000 China; 2 Key Laboratory of Thermal Disaster and Prevention, Ministry of Education, Huludao, 125000 China; 3 Beijing Urban Construction Rail Transit Construction Engineering Co., Ltd., Beijing, 101125 China; 4 Shanxi Jinshen Energy Co., Ltd., Xinzhou, 034000 China

**Keywords:** Spontaneous combustion, Open pit coal pile, Numerical simulation, Water cool steel pipe, Energy science and technology, Materials science

## Abstract

During the storage and transportation process after mining, coal piles are placed in open environments, making them prone to self-heating and spontaneous combustion due to the nature of coal and factors like natural wind flow. In recent years, there have been frequent spontaneous combustion incidents involving coal piles, posing significant safety risks. To effectively prevent and control spontaneous combustion disasters in open-air coal storage piles, we propose a method involving the arrangement of water-cooling steel pipes within the coal piles. This method applies theories of coal spontaneous combustion mechanisms, porous media heat transfer, and non-isothermal pipeline heat transfer. The multi-physics coupling model of COMSOL numerical simulation software is used to analyze the spontaneous ignition process and prevention effect of open pit coal pile. In the model, the thin material transfer of porous media is taken as the oxygen concentration field, the heat transfer of porous media is taken as the temperature field, and the free and porous media flow is taken as the air seepage velocity field. The simulation results of the spontaneous combustion process in the coal pile indicate that the high-temperature zone of spontaneous combustion is situated within the range of 0.5 ~ 1.5 m inside the wind-facing surface and extends 0.5 m above the ground level. These findings serve as a basis for determining the optimal placement of water-cooling steel pipes within the coal pile. The simulation results of a single water-cooling steel pipe demonstrate a positive correlation between the cooling effect on the coal pile and the water cool flow, and a negative correlation with the water cool temperature. Additionally, the cooling radius of the water-cooling steel pipe is determined by the circumference of the pipe and remains unaffected by the water cool flow. Finally, simulations were conducted to evaluate the cooling effect of multiple rows of steel pipes, and optimal arrangement parameters were determined: a center distance between steel pipes of 1 m and a water cool flow rate of 1500 L/min. As a result, the onset of the self-heating period in the coal pile was delayed by 11 days, and the spontaneous combustion period was extended by 56 days. The arrangement of water-cooling steel pipes in the coal pile has demonstrated significant efficacy in preventing and controlling spontaneous combustion.

## Introduction

Due to the increasing demand and depletion of shallow coal reserves, coal mine disasters gradually increase with the growth of mining depth^1–3^. Coal spontaneous combustion occurs not only in underground coal mines but also frequently in coal storage and transportation in open-pit mining, mines, power plants, wharves, gathering stations, and other locations^4^. Spontaneous combustion of coal is a common problem in coal mine disasters. For example, coal field fires of varying degrees have occurred in China, South Africa, Australia, and the United States^5–8^. When coal is deposited in a fragmented state, oxidation reactions occur upon contact with oxygen. If the heat storage environment is conducive, the coal temperature will continue to rise, leading to spontaneous combustion accidents in coal piles^9–13^.

Spontaneous combustion of coal piles not only wastes resources but also causes casualties and pollutes the environment, prompting many scholars to conduct research on preventing and controlling this phenomenon. Zhang^14,15^ employs an improved wire-mesh basket test method to establish a two-dimensional(2D) multi-field coupling model considering the aging effect. They study the change in temperature and oxygen concentration in coal depots. Ejlali^16^ studies the heat and fluid flow through coal stockpiles and the surrounding area using numerical simulation. Based on this, they examine the variation of maximum temperature and consequent heat removal phenomena under transient and steady-state conditions. Cheng^17^ establishes a physical–mathematical model of coal-HP-air and investigates the spontaneous combustion process of coal piles, studying the effect of heat pipes (HPs) at four different inclinations on the spontaneous combustion of coal piles. There are two ways to prevent and inhibit the spontaneous combustion of coal: cutting off oxygen and reducing the coal temperature. Currently, various fire prevention and suppression techniques are employed to prevent the spontaneous combustion of coal. These include pressurized grouting gel^18–20^, grouting foam^21–23^, spraying inhibitors^24,25^, and pumping inert gases (CO_2_ and N_2_)^26–28^. However, the aforementioned prevention and control technologies all suffer from limitations such as lengthy treatment times, low efficiency, and high costs. Therefore, finding a high-efficiency and low-cost method to prevent spontaneous combustion and destroy the heat storage state of coal piles has become an urgent issue.

In this paper, the heating position of the coal pile in its natural state is determined using numerical simulation software, and steel pipes are arranged in the high-temperature area. Water is chosen as the heat exchange medium, and the heat stored in the coal body is directly absorbed by the flowing water in the steel pipe to accurately cool the high-temperature area. The distribution of the temperature field of the coal pile before and after the installation of the water-cooling steel pipes is analyzed, and a layout scheme for the water-cooling steel pipes to prevent spontaneous combustion of the coal pile is obtained.

## Determination of basic parameters of coal sample

### Proximate analysis of coal samples

The coal sample used in the experiment was taken from a coal mine in Shanxi Province. The 5E-MAG6700 industrial analyzer was used to analyze the coal sample and measure its moisture, ash, and volatile matter content. The results are shown in Table 1: The coal sample has low moisture content but high volatile content, making it prone to low-temperature oxidation and spontaneous combustion phenomena.

**Table 1:** Proximate analysis of coal sample.

Proximate analysis			Total sulfur	True relative density	Quantity of respiratory oxygen (cm^3^/g dry coal)
Moisture%	Ash content%	Volatile matter%			
1.76	17.44	43.64	0.61	1.46	0.72

Based on the results of oxygen absorption and industrial analysis, the evaluation of the coal's spontaneous combustion tendency grade is determined, as shown in Table 2.

**Table 2:** Evaluation of coal spontaneous combustion tendency grade when coal sample oxygen absorption V_d_ > 18%.

Grade of spontaneous combustion tendency	Spontaneous combustion tendency	Oxygen absorption of coal V_d_ (cm^3^/g dry coal)
I	Easy spontaneous combustion	V_d_ > 0.7
II	Self-ignite	0.4 < V_d_ ≤ 0.7
III	Not easy to spontaneous combustion	V_d_ ≤ 0.4

Based on the results of industrial analysis and the spontaneous combustion tendency grade of the coal sample, it is classified as Class I, indicating a high susceptibility to spontaneous combustion.

### Thermogravimetric analysis experiment

**Figure 1:**
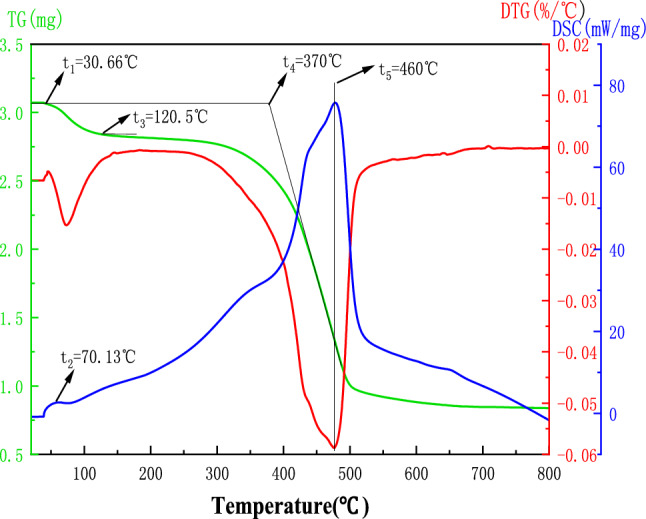
TG/DTG curves of oxidized coal samples.

The thermogravimetric analysis of coal samples was conducted using a synchronous thermal analyzer. The coal samples consisted of pulverized coal with a particle size less than 0.125 mm and a mass of 3.07 mg. They were heated from 20 °C to 800 °C at a rate of 10 °C/min in a dry air atmosphere. The TG/DTG curve of the coal sample is shown in Fig. 1.

Based on the TG/DTG curve of coal samples, the following characteristic temperature points are obtained according to Fig. 1: the critical temperature of spontaneous combustion of coal T_1_(343 K), the dry cracking temperature T_2_(393 K), and the ignition point temperature T_3_(623 K). The spontaneous combustion process of coal is divided into four stages: the latent period, the self-heating period, the spontaneous combustion period, and the gas stove phase. These characteristic temperature points provide parameters for the subsequent numerical simulation process.

## Construction of coal pile and water-cooling steel pipe model

### Cooling principle of water-cooling steel pipe

**Figure 2:**
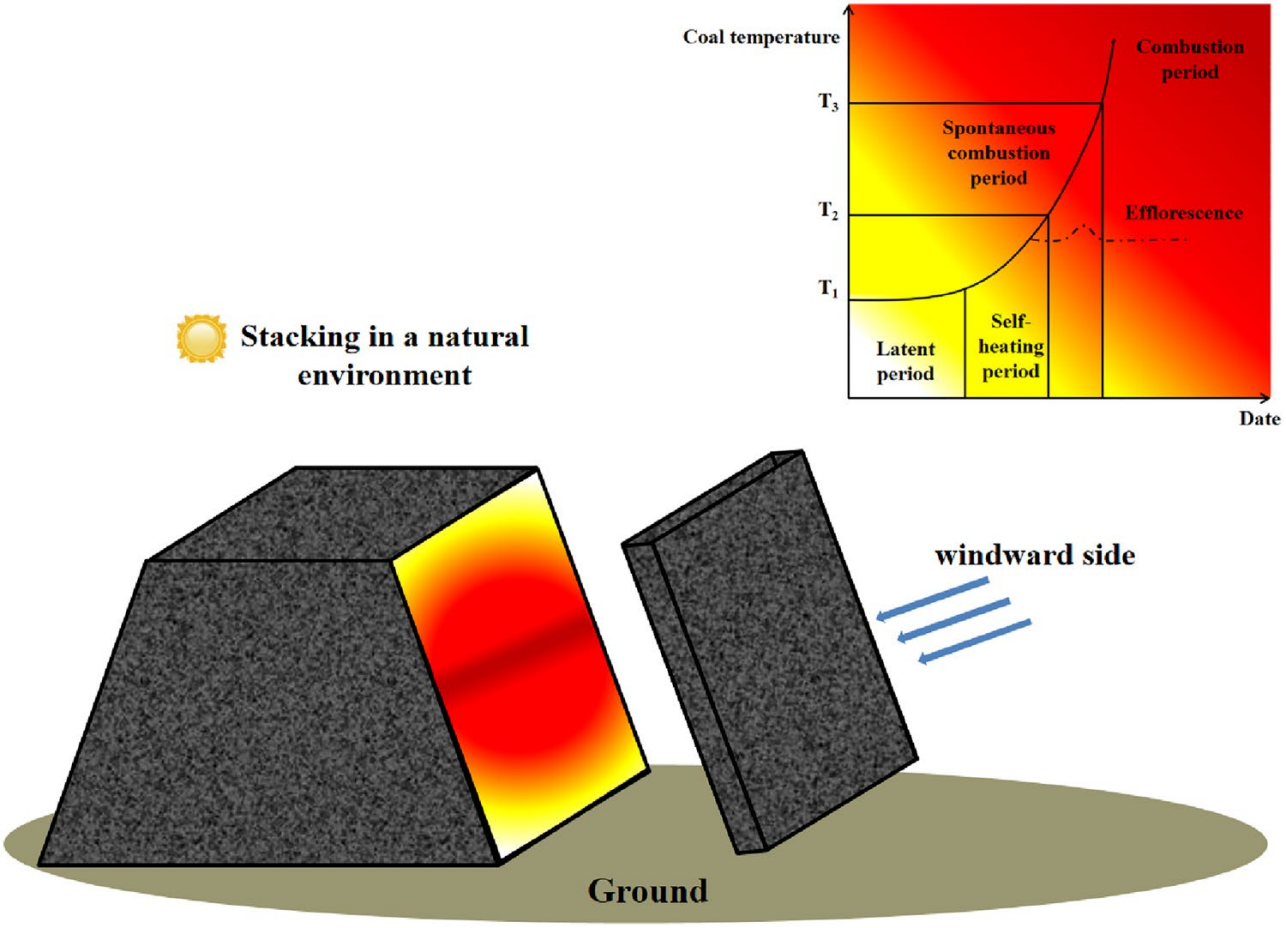
Schematic diagram of spontaneous combustion of open-air coal pile.

Figure 2 shows the spontaneous combustion process of coal pile in natural position.

With the increase in the temperature of the coal pile, the high-temperature region of the coal pile will shift towards the surface where there is sufficient oxygen supply and favorable heat storage conditions. Oxygen-depleted conditions can reduce the heat release of coal, delaying the occurrence of characteristic temperature points and prolonging the reaction time^29^. Controlling the temperature of the coal pile during the self-heating period or below can prevent spontaneous combustion. Therefore, the temperature range between T_1_ and T_2_ is an important stage in preventing spontaneous combustion of the coal pile.

**Figure 3:**
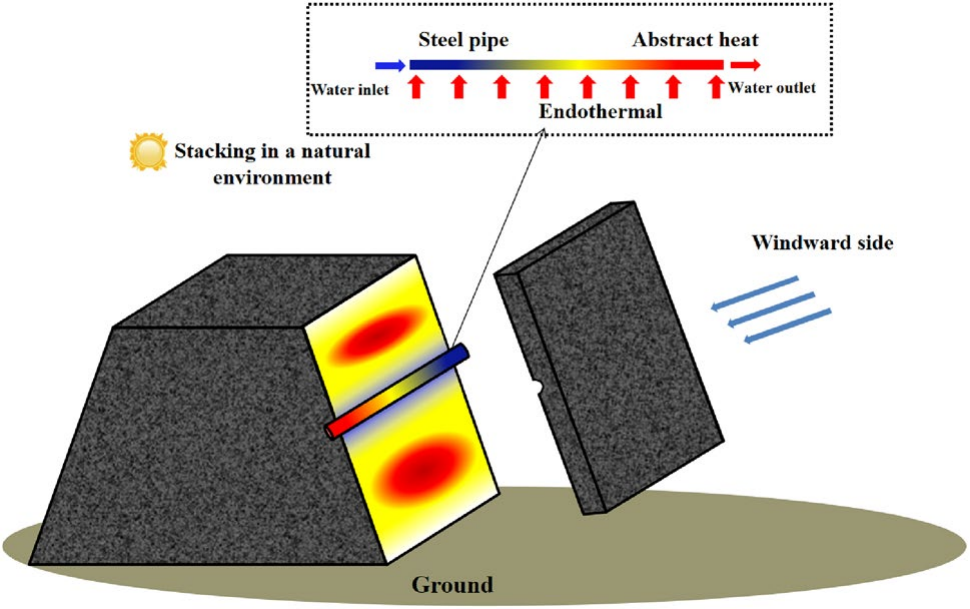
Schematic diagram of water injection steel pipe cooling.

As depicted in Fig. 3, the flowing water in the water-cooling steel pipe can effectively dissipate the heat stored in the coal pile, thus achieving the purpose of cooling it. Therefore, based on the size and duration of the coal pile's stacking, a layout scheme for the water-cooling steel pipe in the high-temperature area of the coal pile is designed in advance. This enables precise cooling of the coal pile during the self-heating period (343 K ~ 393 K) and extends its ignition time.

### Coal spontaneous combustion model

The multi-physics field coupling model of COMSOL Multiphysics software is utilized to simulate the spontaneous combustion process of the coal pile^30^. In this model, dilute matter transfer in porous media represents the oxygen concentration field, heat transfer in porous media represents the temperature field, and free and porous media flow represents the air seepage velocity field.

#### Basic equation

1. Air seepage velocity field equation.
  The air flow inside the coal pile satisfies Brinkman equation^31,32^, i.e.:
  1 $$- \nabla p = \frac{\mu }{k}v - \mu \nabla^{2} v$$
  where, ∇ is the Hamilton operator; *p* is pressure, Pa; *μ* is dynamic viscosity, Pa·s; *k* is the permeability coefficient of coal pile; *v* is the velocity vector, m/s.
2. Oxygen concentration field equation.
  Oxygen flows in the pores of coal body and redox reaction occurs with coal body. The equation of oxygen concentration change is:
  2 $$n\frac{\partial c}{{\partial t}} + v\nabla c = D\nabla^{2} c - \left( {1 - n} \right)r$$
  where, *n* is coal porosity, %; *c* is oxygen concentration, mol/m^3^; *t* is time, s; *D* is the air diffusion coefficient, m^2^/s; *r* is the oxygen consumption rate, mol/(m^3^·s).
3. Coal pile temperature field equation.
  Heat transfer between gas phase and solid phase in coal pile satisfies the heat balance hypothesis^33^, the expression is:
  3 $$\left[ {\left( {n\rho_{g} C_{g} } \right) + \left( {1 - n} \right)\rho_{s} C_{s} } \right]\frac{\partial T}{{\partial t}} + \left( {v\nabla } \right)\left( {\rho_{g} C_{g} T} \right) = k_{1} \nabla^{2} T + Q + Q_{c}$$
  where, *ρ*_*g*_ and *ρ*_*s*_ respectively represent the density of air and coal body, kg/m^3^; *C*_*g*_ and *C*_*s*_ represent the specific heat capacity of air and coal, J/(kg·K); *k*_*1*_ is the thermal conductivity between air and coal, W/(m·K); *Q* is the heat release intensity of coal, W/m^3^. *Q*_*c*_ is the heat lost from coal to air, W/m^3^.
  The expression of heat loss from coal to air is:
  4 $$Q_{c} = h\left( {T - T_{i} } \right)dx$$
  where, h is the heat transfer coefficient, W/(m^2^·K); Ti is the initial temperature of coal pile, K; dx is the unit length of the contact surface between coal and air, m.

The number of heat transfer coefficient is determined by the specific size of the coal pile and the way of convection with the air.

#### Coal pile parameter

In the natural environment, the wind speed naturally flows into the coal body. However, due to seepage losses, the wind speed decreases to approximately zero within the coal pile. Therefore, the wind speed for air seepage inside the coal pile is set to *v* = 0.0001 m/s. The initial temperature of the coal pile and the external air temperature are both set to 300.15 K. The main parameters of the coal pile model are obtained from the coal sample test results of a coal mine in Shanxi, as shown in Table 3.

**Table 3:** Model calculation parameters.

Parameter	Physical meanings	Parameter values
*ρ*_*g*_	Coal body density (kg/m^3^)	1400
*ρ*_*s*_	Air flow density (kg/m^3^)	1.16
c_i_	Initial oxygen concentration (mol/m^3^)	9.375
*C*_s_	Coal body specific heat capacity (J/(kg· K))	1200
*C*_g_	Air specific heat capacity (J/(kg· K))	1005
*k*_*1*_	Thermal conductivity (W/ (m· K))	0.2
*D*_*0*_	Initial oxygen diffusivity (m^2^/s)	1.50 × 10^–5^
*d*_*50*_	Median particle size of coal in a coal pile (mm)	20
*p*_*0*_	Initial atmospheric pressure (atm)	1
*n*	Accumulation porosity	0.5
*k*	The permeability coefficient of the coal stacks	4.66 × (10^–4^) × 10^–1.69/n^

The formula of oxidation heat release rate of coal sample is as follows:

5 $$Q = \left\{ {\begin{array}{*{20}c} {6.021 \times 10^{10} \times \left[ {0.9503 - 0.1286 \times \ln \left( {\frac{{d_{50} }}{6.5}} \right)} \right] \times \frac{c}{{c_{i} }} \times \exp \left( {\frac{ - 6231.8}{T}} \right)} & {273.15{\text{K}} \le T \le 343.15{\text{K}}} \\ {6.021 \times 10^{10} \times \left[ {0.5602 - 0.3986 \times \ln \left( {\frac{{d_{50} }}{6.5}} \right)} \right] \times \frac{c}{{c_{i} }} \times \exp \left( {\frac{ - 6231.8}{T}} \right)} & {T \ge 343.15{\text{K}}} \\ \end{array} } \right.$$

The oxygen consumption rate of the coal sample is as follows^34^:

6 $$r = \left\{ {\begin{array}{*{20}c} {1.86 \times 10^{ - 3} \times \left[ {1.365 - 0.983 \times \ln \left( {\frac{{d_{50} }}{6.5} + 1.585} \right)} \right] \times \frac{c}{{c_{i} }} \times \exp \left( {\frac{ - 4646}{T}} \right)} & {273.15{\text{K}} \le T \le 343.15{\text{K}}} \\ {1.86 \times 10^{ - 3} \times \left[ {0.659 - 0.592 \times \ln \left( {\frac{{d_{50} }}{6.5} + 0.183} \right)} \right] \times \frac{c}{{c_{i} }} \times \exp \left( {\frac{ - 4646}{T}} \right)} & {T \ge 343.15{\text{K}}} \\ \end{array} } \right.$$

The oxygen diffusion coefficient is shown below:

7 $$D = \left( {0.8011 \times n - 0.1616} \right) \times D_{0} \times \left( {\frac{T}{{T_{i} }}} \right)^{\frac{2}{3}}$$

where, *T* is the temperature of coal pile, K; *T*_*i*_ is the initial temperature of the coal pile, K.

#### Initial boundary condition

The heat source of the model is the oxidation reaction that occurs between coal and oxygen. Oxygen enters from the windward side, causing continuous oxidation of the coal upon contact and releasing heat. Regarding heat dissipation, the primary mechanism involves heat conduction within the coal body and interactions with the coal wall surface. Any remaining accumulated heat is then exchanged with the water-cooling steel pipe. As water flows at a certain speed, heat generated inside the coal pile is removed through exchange with the flowing water, thereby achieving the purpose of cooling.

The YOZ direction of the model is set as the wind direction of the coal pile, that is, the inlet of the air flow, and the other faces are all the outlet of the air flow. The initial temperature of the coal pile is 300 K, the same as that of the atmospheric environment, and the specific boundary conditions are shown in Table 4.

**Table 4:** Initial boundary conditions.

Physical field	Boundary condition	
	Entrance	Exit
Porous medium for dilute substance delivery	$c\left| {_{t = 0} = c_{0} } \right.$ Initial oxygen concentration on the exposed side	$- nD\nabla c_{i} - = 0$ Gas flow on the remaining side
HEAT transfer in porous media	$T\left| {_{t = 0} = T_{0} } \right.$ Initial temperature of coal body	$Q = h_{s} \left( {T_{0} - T} \right)$ Side heat flux of the coal pile
Free and porous medium flow	$v\left| {_{t = 0} = v_{0} } \right.$ Wind speed on the wind side	$p = p_{0}$ Free flow of gas
Unsteady state temperature pipe flow	$q_{m,0} = Q_{w}$ Rate of flow of fluid	

### Steel pipe cooling model

#### Equation of physical field inside steel pipe

In a steel tube, the flow of water is represented by the momentum and mass conservation equations:

8 $$\rho \frac{\partial u}{{\partial t}} = - \nabla p - f_{D} \frac{\rho }{{2d_{h} }}u\left| u \right|$$

9 $$\frac{\partial A\rho }{{\partial t}} + \nabla \cdot \left( {A\rho u} \right) = 0$$

where, *u* is the average fluid velocity of the cross section in the tangential direction of the pipe center line, m/s; *A* is the cross-sectional area of the pipeline, m^2^; *ρ* is the fluid density, kg/m^3^.

#### Determine water-cooling pipe parameters

Table 5 shows the related parameters of water-cooling steel pipes.

**Table 5:** Water-cooling steel pipe parameter.

Parameter	Physical meanings	Parameter values
*T*_e_	Initial water temperature (K)	300.15
*d*	Steel pipe outer diameter (mm)	125
*d*_1_	Steel pipe inner diameter (mm)	100
*e*	Surface roughness (mm)	0.046

### Geometric model

**Figure 4:**
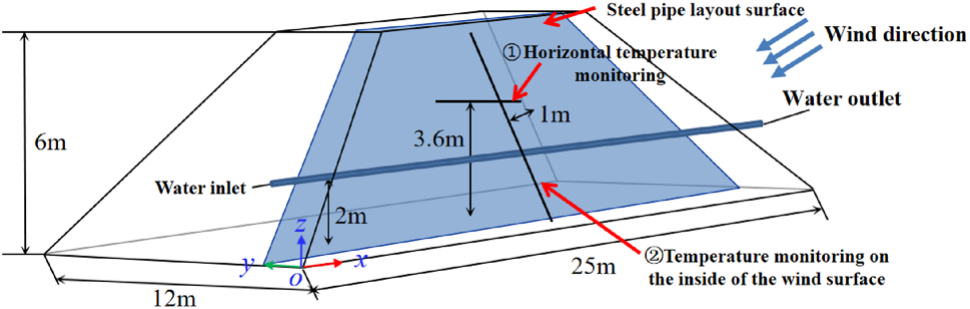
Coal pile geometry.

The initial size of the open-pit coal pile is set to 25 × 12 × 6 m, with the plane YOZ representing the windward side, as depicted in Fig. 4. When the coal pile is situated in a sufficiently large atmospheric environment area, airflow disturbances around the coal pile tend to stabilize, and the inlet and outlet airflows converge^35^.

### Grid construction

**Figure 5:**
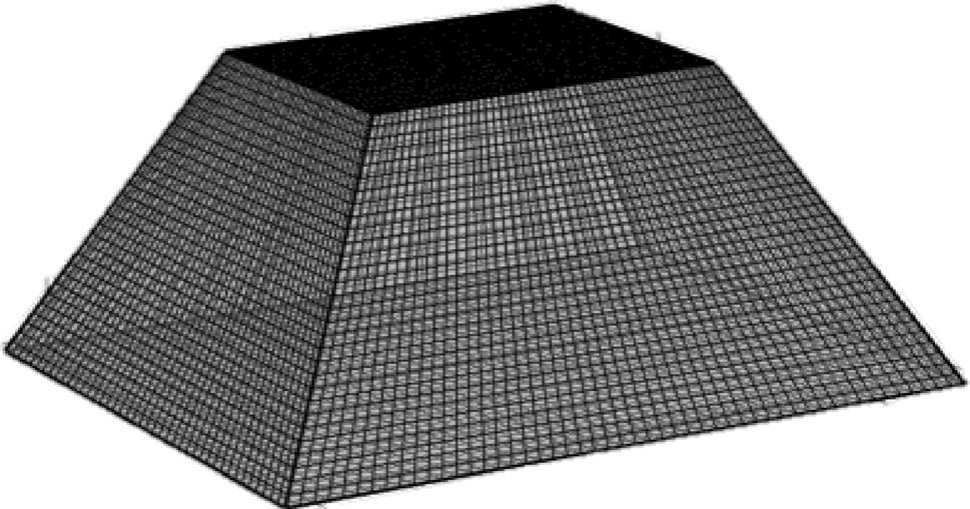
Model Mesh Construction Diagram.

In order to improve the accuracy of numerical analysis, the established geometric model is grid-divided. The grid section of coal pile and water-cooling pipe is shown in Fig. 5. The number of peak units: 8; boundary units: 320; unit: 75,825; minimum unit mass: 0.04138.

## Analysis of cooling performance of water-cooling steel pipe

### Determine the location of water-cooling steel pipe

**Figure 6:**
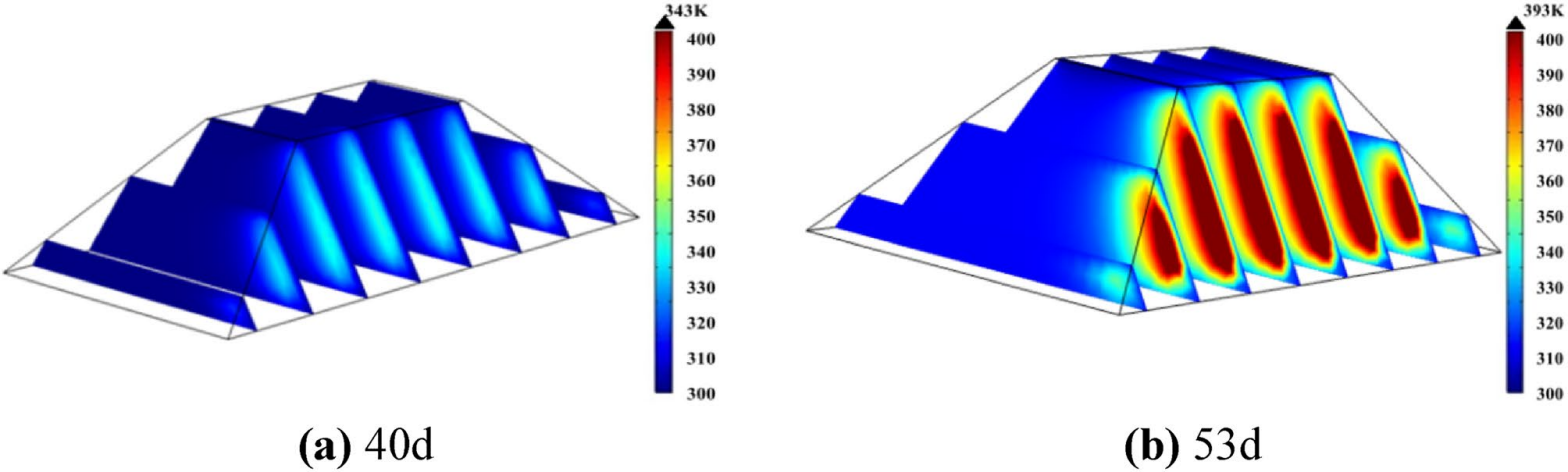
Internal temperature of coal pile naturally placed.

In order to determine the range of high temperature zone, firstly, numerical simulation of spontaneous combustion of coal pile is carried out, and the results are shown in Fig. 6.

According to Fig. 6a, after 40 days of natural stacking, the maximum temperature inside the coal pile reaches 343 K, indicating the onset of the self-heating stage. As shown in Fig. 6b, after 53 days of natural stacking, the maximum temperature inside the coal pile reaches 393 K, signaling the onset of spontaneous combustion. Additionally, Fig. 4 illustrates that the high-temperature zone of the coal pile is parallel to the wind surface and gradually diffuses towards the surface.

**Figure 7:**
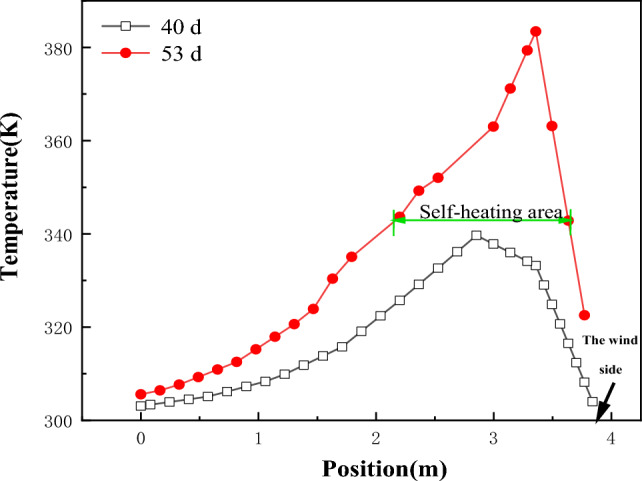
The internal temperature change diagram in the X direction at the origin of the coal pile.

Figure 7 displays the temperature variation of the coal pile at a height of 3.6 m along the X direction toward the windward side, with its monitoring position indicated by the "① Horizontal temperature monitoring line" in Fig. 4. According to Fig. 7, the highest temperature inside the 40-day-old coal pile occurs 1.15 m away from the windward side, while in the 53-day-old coal pile, the highest temperature gradually shifts outward to 0.5 m away from the windward side. Furthermore, the coal within the horizontal depth range of 0.5 m to 1.15 m from the windward surface undergoes self-heating between 40 and 53 days.

**Figure 8:**
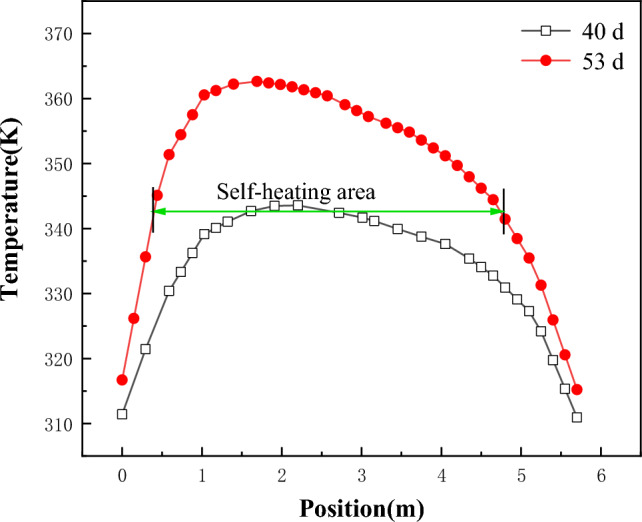
Temperature variation diagram of coal pile parallel to the wind-receiving surface at a depth of 1 m.

Monitor the temperature change at a depth of 1 m from the coal pile to the windward surface. The monitoring position is shown in Fig. 4, labeled as "② Temperature monitoring on the inside of the wind surface," and the monitoring results are shown in Fig. 8. As can be seen from Fig. 8, between the 40th and 53rd days, the highest temperature inside the coal pile reaches 2 m in height. By the 53rd day, the coal within the vertical range of 0.5 m to 5 m is in the self-heating stage.

Based on the research results mentioned above, it is determined that the coal pile self-heating area extends within the range of 0.5 to 1.15 m from the windward side in the horizontal direction and 0.5 to 5 m in the vertical direction. Therefore, design and arrangement should be made within this range.

### Determine the cooling range and radius of a single steel pipe

According to the simulation results mentioned above, the steel pipe is arranged in the high-temperature section on the windward side, positioned 2 m above the ground and 1 m from the windward side, as illustrated in Fig. 4. The water-cooling steel pipe is set to flow when the maximum temperature inside the coal pile reaches 343 K, and temperature changes inside the coal pile are monitored.

#### Influence of flow rate on cooling range and radius

The flow rate of the water-cooling steel pipe directly affects its heat transfer performance. To determine the influence of flow rate on the cooling range and radius, simulation experiments were conducted using single steel pipes with flow rates of 0, 100 L/min, 300 L/min, 500 L/min, 700 L/min, and 900 L/min.

**Figure 9:**
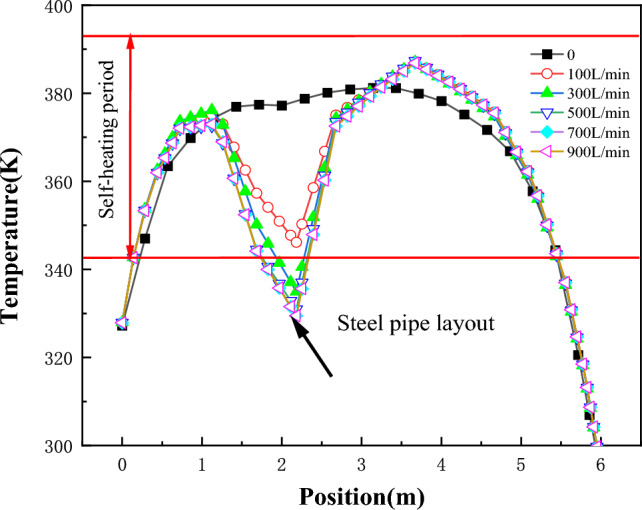
The temperature change diagram of the arrangement surface of the steel pipe on the 60th day with different flow rates.

**Figure 10:**
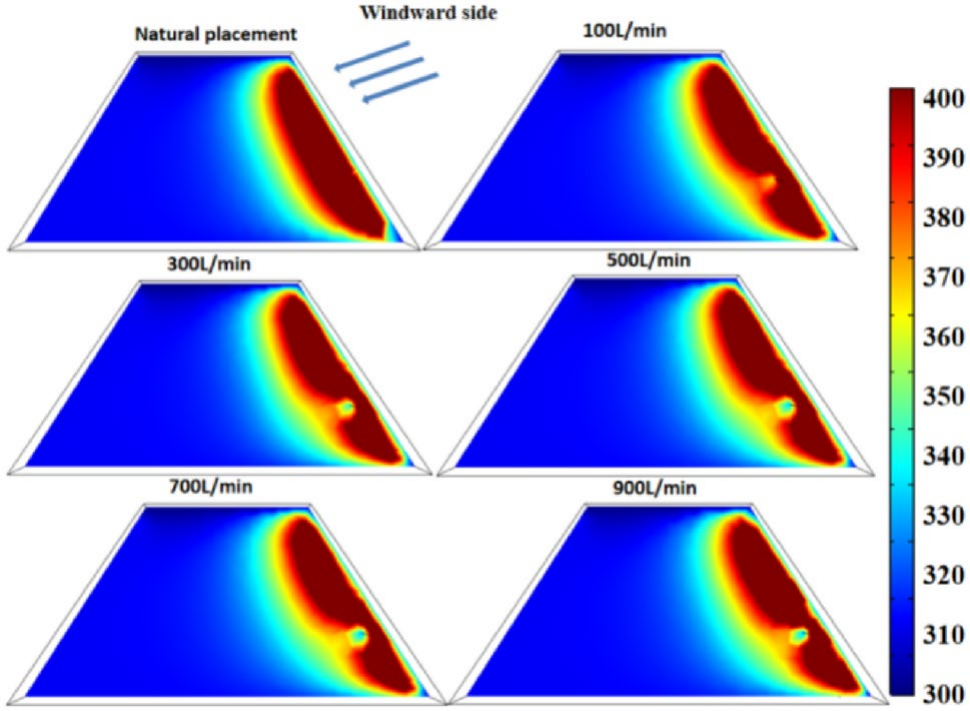
Comparison chart of temperature change of coal pile before and after water injection of steel pipes with different flow rates.

Figure 9 displays the temperature variation of "temperature monitoring line ②"at different flow rates, while Fig. 10 illustrates the temperature field variation of the coal pile before and after the layout of water-cooling steel pipes.

It can be observed from Figs. 9 and 10 that the cooling range of the water-cooling steel pipe is positively correlated with the flow rate. When the flow rate exceeds 500 L/min, the minimum coal temperature drops to 322 K. Compared to a flow rate of 0, this represents a temperature reduction of 55 K, and compared to the absence of steel pipe layout, the temperature drops by 80 K.

**Figure 11:**
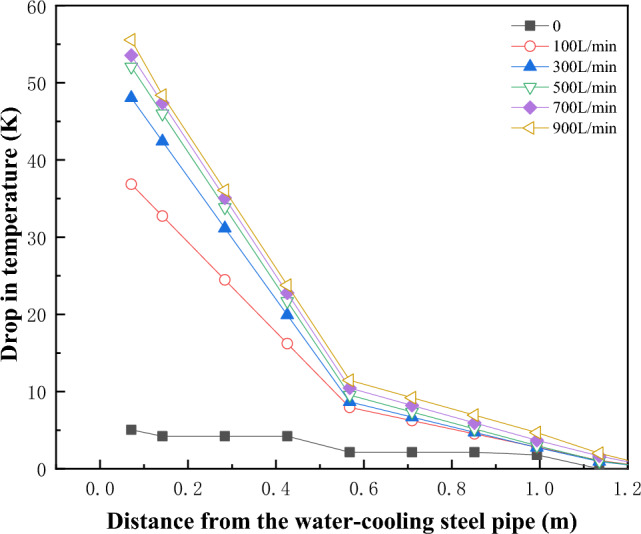
The cooling rate inside the coal pile near the water-cooling steel pipe.

Figure 11 shows the temperature change of coal pile at the 60d water-cooling steel pipe.

As seen from Fig. 11, the greater the flow rate, the larger the cooling range. However, when the flow rate exceeds 500L/min, there is little difference in both the cooling range and radius, suggesting that further increasing the flow rate may not be significantly beneficial. Additionally, Fig. 11 illustrates that regardless of the flow rate, the cooling effect is relatively significant within a 0.5 m distance from the water-cooling steel pipe.

#### Influence of water temperature on cooling range and radius

The water temperature of the inlet will affect the heat transfer between the fluid in the pipe and the coal pile. To study the influence of the inlet water temperature on the cooling effect of the water-cooling steel pipe, comparative experiments are conducted with inlet water temperatures of 300 K, 310 K, 320 K, 330 K, and 340 K respectively.

**Figure 12:**
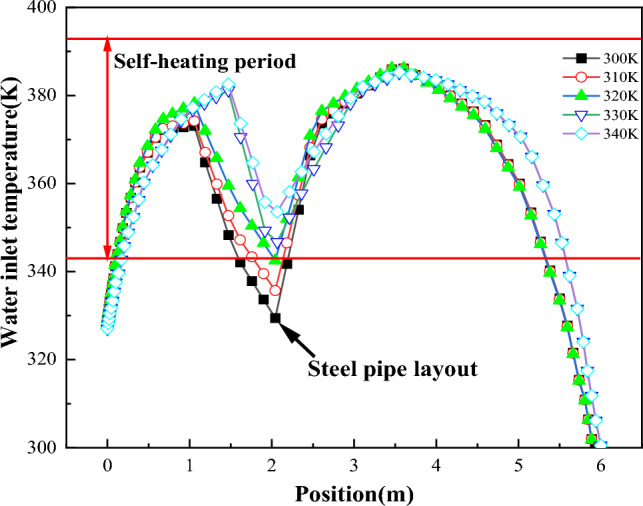
Variation diagram of coal pile temperature on the 60th day with different cooling water temperatures.

**Figure 13:**
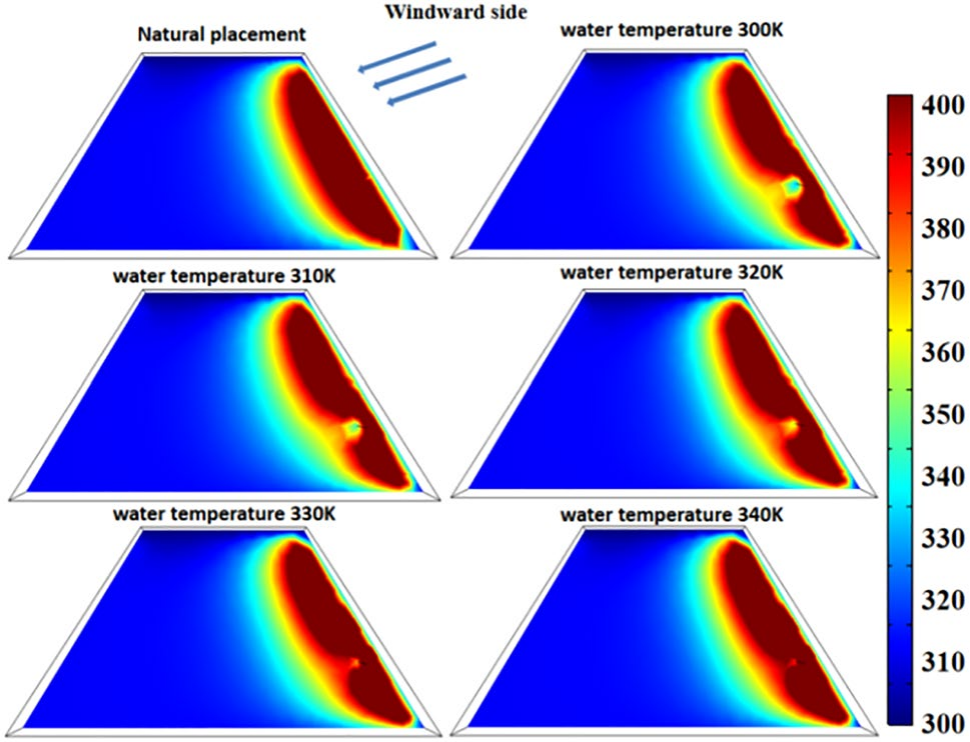
Comparison of temperature changes of coal piles on the 60th day with different cooling water temperatures.

Figure 12 depicts the temperature change along temperature monitoring line ② with varying water temperatures at the inlet of the water-cooling steel pipe. Figure 13 illustrates a comparison of the temperature change in coal piles over 60 days under different inlet water temperatures. It can be observed from Figs. 12 and 13 that the inlet water temperature significantly influences the heat exchange performance of the water-cooling steel pipe. Lower inlet water temperatures result in greater reductions in coal temperature around the steel pipe. For instance, compared to a water temperature of 340 K, the maximum coal temperature of the same coal pile is 33 K lower when the water temperature is 300 K.

#### Analysis of simulation results of a single steel pipe

1. Cooling radius of water-cooling steel pipe.
  As shown in Fig. 14: the heat transfer mode through the tube wall satisfies the following formula:
  10 $$Q_{w} = hZ\left( {T - T_{{\text{e}}} } \right)$$
  where, *Q*_*w*_ is heat transfer over meters, W/m; *Z* is the circumference of steel pipe, m; *h* is the heat transfer coefficient of the fluid in the tube, W/(m^2^·K); *T* is the coal temperature near the wall, K; *T*_*e*_ is the cooling water temperature, K.

**Figure 14:**
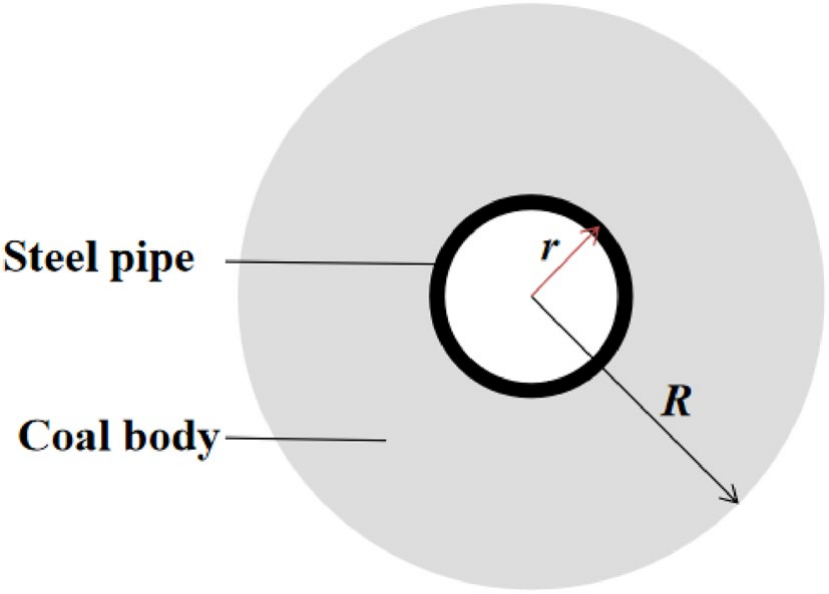
Schematic diagram of cooling radius of steel pipe.
  The heat absorbed by steel pipe per unit length is:
  11 $$Q_{1} = Q_{w} dx$$
  where, *Q*_*1*_ is the heat absorbed by the tube wall, W; *dx* is the unit length of the steel pipe, m.
  Equation^36^ can be used to calculate the influence range of water-cooling steel pipe on coal temperature within a unit time:
  12 $$Q_{2} = q\left( {\pi R^{2} - \pi r^{2} } \right)dx\rho_{g} /dt$$
  where, *Q*_*2*_ is the heat released by coal body, W; *q* is the calorific value of coal, J/kg, *R* is the cooling radius of steel pipe to coal, m; *r* is the outer radius of the steel pipe, m; *ρ*_*g*_ is the density of coal, kg/m^3^; *dt* is unit time, s.
  When *Q*_*1*_=*Q*_*2*_, heat transfer efficiency reaches the maximum and cooling radius also reaches the maximum.
  13 $$Q_{w} dx = q\left( {\pi R^{2} - \pi r^{2} } \right)dx\rho_{g} /dt$$
  Therefore
  14 $$R{ = }\sqrt {\frac{{2hr\left( {T - T_{e} } \right)dt}}{{q\rho_{g} }} + r^{2} }$$
  It can be seen from Eq. (14) that the cooling radius (*R*) of the water-cooling steel pipe is related to the heat transfer coefficient of water (*h*), the heat value of coal (*q*) and the outer diameter of the steel pipe (*r*), and has nothing to do with the velocity of water. When the steel pipe parameters in the model are used, the cooling radius of the water-cooling steel pipe is 0.5 m.
2. The influence of flow rate on temperature drop amplitude.
  Increasing the inlet flow rate will increase the cooling range of the steel pipe, but with the continuous increase of the flow rate, the cooling range of the steel pipe will grow smaller and smaller. When the pipe diameter is constant, the relationship between flow rate and heat transfer power can be obtained according to formula (10), as shown in Fig. 15.

**Figure 15:**
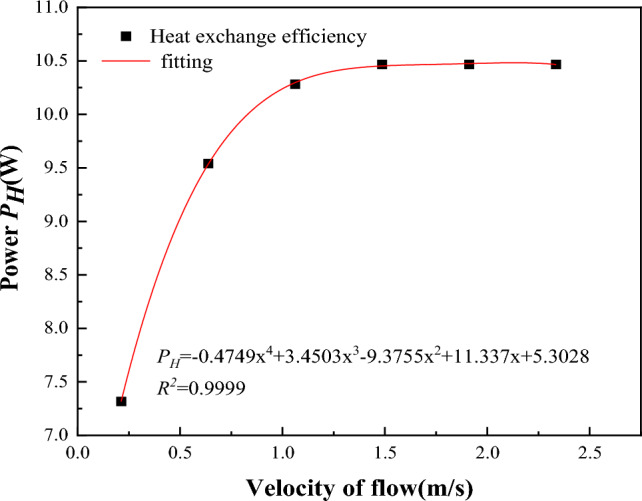
Flow rate-heat transfer power fitting curve.
  The relationship between velocity and heat transfer power can be obtained as follows:
  15 $$P_{H} = - 04749v^{4} + 3.4503v^{3} - 9.3755v^{2} + 11.337v + 5.3028$$
  where, v is the velocity of water, m/s; PH is the heat transfer power.
  As can be seen from Fig. 15, the heat transfer power increases with the increase of the flow velocity. When the flow velocity increases to a certain value, the heat transfer power does not increase when it reaches the maximum value, and the flow velocity at this time (500L/min) is the optimal flow velocity under this condition.
3. The influence of initial water temperature on the range of cooling.
  The lower the inlet temperature, the greater the temperature difference between the water temperature in the pipe and the surrounding coal body, the more cold water transferred to the coal body in the pipe. The endothermic formula of water is:
  16 $$Q = cm\left( {T_{2} - T_{1} } \right)$$
  where, *Q* is the heat absorbed by water when it heats up, J; *c* is the specific heat capacity of water, J/(kg·K); *m* is the mass, kg; *T*_*2*_ is the outlet water temperature, K; *T*_*1*_ is the inlet water temperature, K.
  As can be seen from the formula, the higher the initial water temperature, the less heat absorbed by the water and the smaller the cooling range. Therefore, when cooling the water supply pipe, the lowest water temperature should be selected.

### Analysis of cooling performance of multi-row water-cooling steel pipe

After determining the cooling performance of a single water-cooling steel pipe, simulation analysis of multiple rows of water-cooling steel pipes was conducted. The parameters used in the simulation are as follows: when the inlet temperature is 300 K, water in the pipe starts to flow when the maximum temperature in the coal pile reaches 343 K, and the center distance between adjacent steel pipes is 1 m.

**Figure 16:**
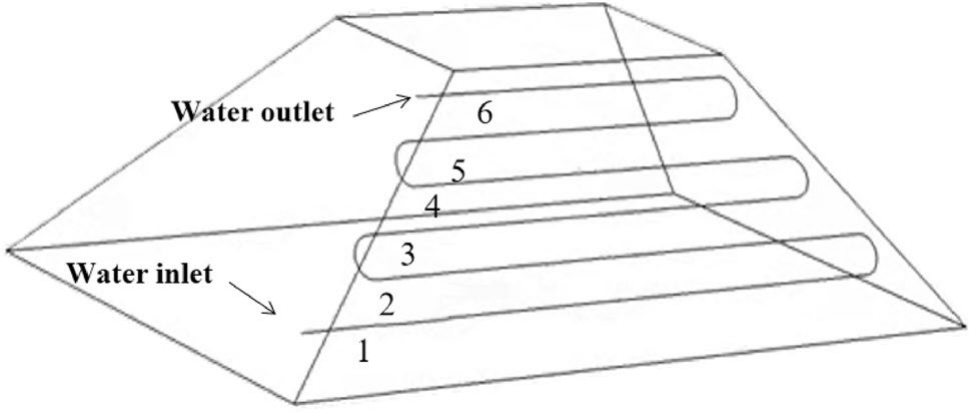
Arrangement of multi-row water-cooling steel pipes.

The cooling simulation of multi-row water-cooling steel pipes with a flow rate of 500L/min was conducted. The steel pipes are arranged in the high-temperature area, which is on a plane parallel to the wind surface at a depth of 1 m, as shown in Fig. 16. According to the simulation results of spontaneous combustion of the coal pile, the temperature of the high-temperature zone of the coal pile is relatively higher under the wind surface. Therefore, the bottom of the coal pile is set as the water inlet, and the top is set as the water outlet.

**Figure 17:**
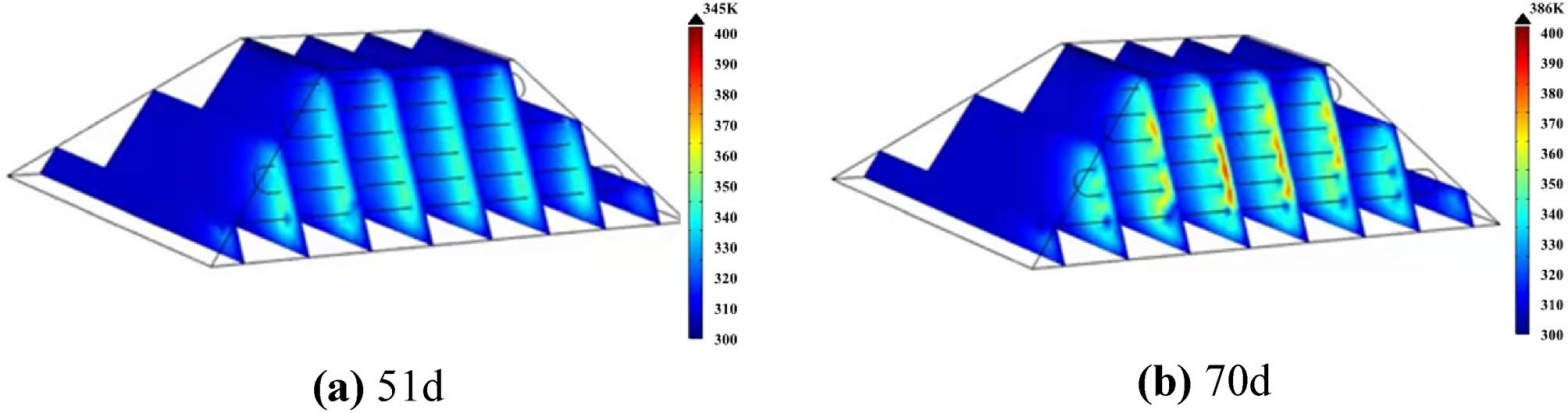
The temperature distribution of coal pile of arrangement of multi-row water-cooling steel pipes.

A 70-day multi-row water-cooling steel pipe cooling simulation was conducted, and the simulation results are shown in Fig. 17. Figure 17a displays the temperature distribution characteristics after multiple rows of water-cooling steel pipes were arranged on day 51. As seen from the figure, after the arrangement of multiple rows of water-cooling steel pipes, the maximum temperature of the coal pile reached 345 K on the 51st day after placement, which is 11 days longer than that without the arrangement of water-cooling steel pipes. At this point, the coal pile enters the self-heating zone, and the water inside the steel pipe begins to flow.

Figure 17b shows the temperature distribution characteristics of coal pile 70d. As can be seen from the figure, after the arrangement of multiple rows of water-cooling steel pipes, the maximum temperature of the coal pile on the 70th day is 386 K, which is 27d longer than that without the arrangement of steel pipes, and the cooling effect is very obvious.

**Figure 18:**
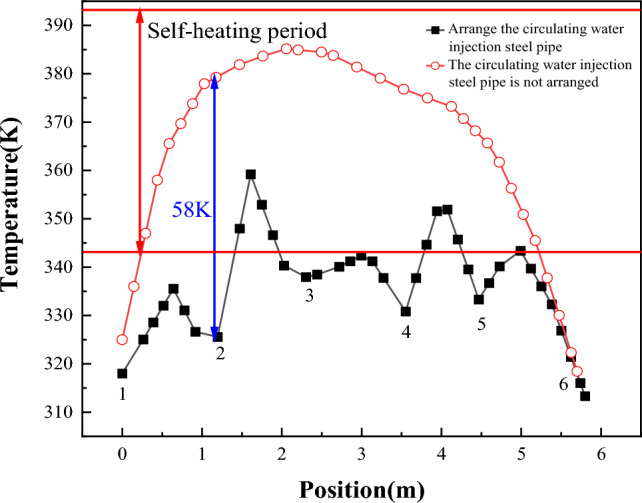
Temperature changes before and after arranging multiple rows of water-cooling steel pipes.

**Figure 19:**
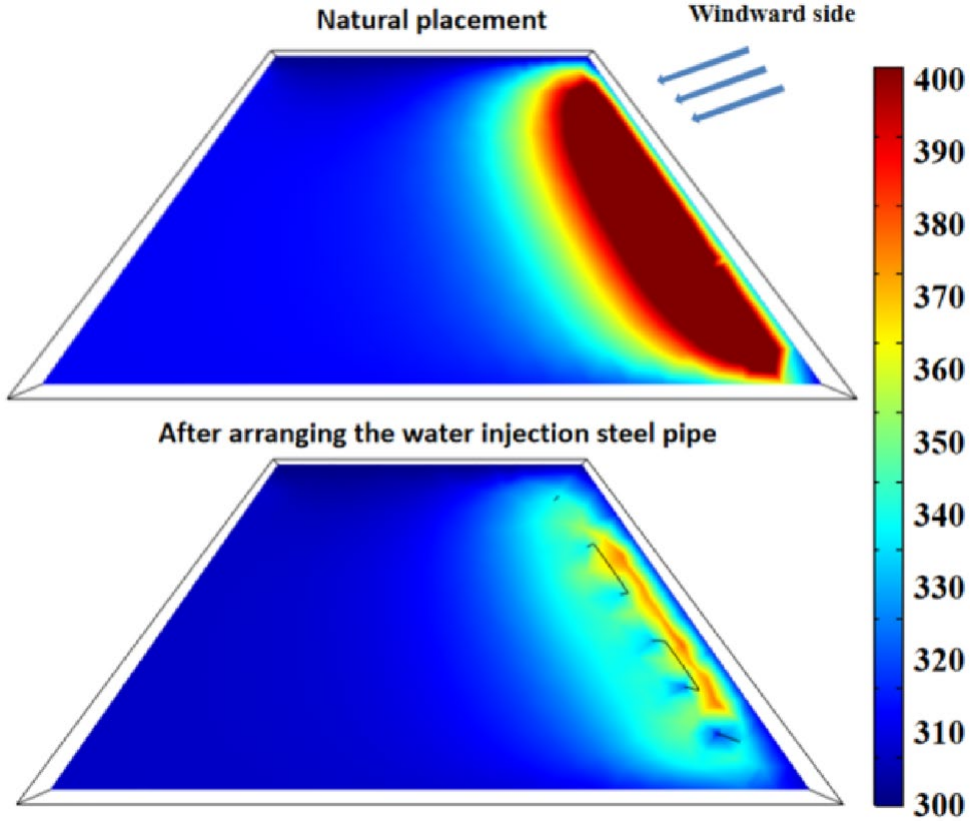
Comparison of coal pile temperatures before and after arranging multiple rows of water-cooling steel pipes.

Figure 18 shows the temperature change of "temperature monitoring line ②" before and after the arrangement of multiple rows of water-cooling steel pipes on the 70th day. The figure shows the position of steel pipes. Figure 19 shows the comparison of coal pile temperature before and after the arrangement of multiple rows of water-cooling steel pipes. It can be seen from Figs. 18 and 19 that, compared with without steel pipes, the coal temperature after steel pipes are arranged is greatly reduced, and the maximum temperature difference before and after the same position reaches 58 K.

**Figure 20:**
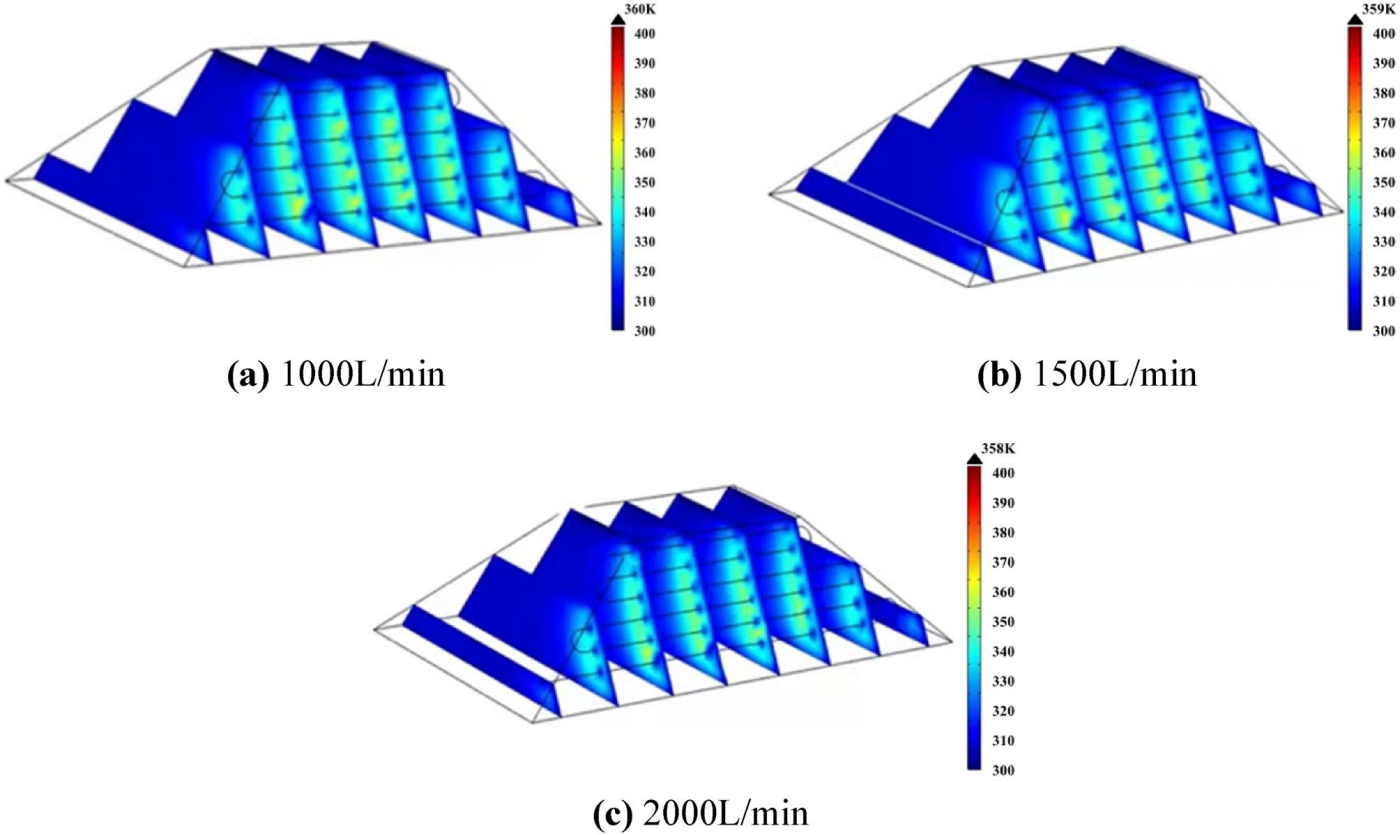
Temperature distribution map of coal pile with different flow rates.

Different from the single water-cooling steel pipe, the full length of the multi-row water-cooling steel pipe is longer, and the heat exchange time between the cold water and the coal body in the steel pipe also becomes longer. In order to ensure the cooling effect, the flow should be appropriately increased according to the simulation results. Therefore, cooling experiments with flow rates of 1000 L/min, 1500 L/min and 2000 L/min were carried out, and the results are shown in Fig. 20.

Figure 20a shows the temperature distribution diagram of the coal pile at the 70th day when the flow rate is 1000 L/min. At this time, the maximum temperature inside the coal pile is 360 K, which is 26 K lower than that at the flow rate of 500 L/min. Figure 20b and c show the temperature distribution of the coal pile at the 70th day when the flow rate is 1500 L/min and 2000 L/min respectively. The maximum temperature of the coal pile is 359 K and 358 K respectively.

**Figure 21:**
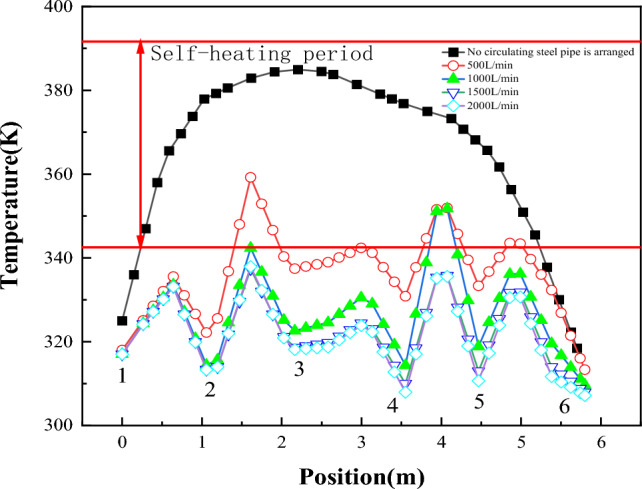
Temperature change on the 70th day before and after the arrangement of multiple rows of water-cooling steel pipes.

**Figure 22:**
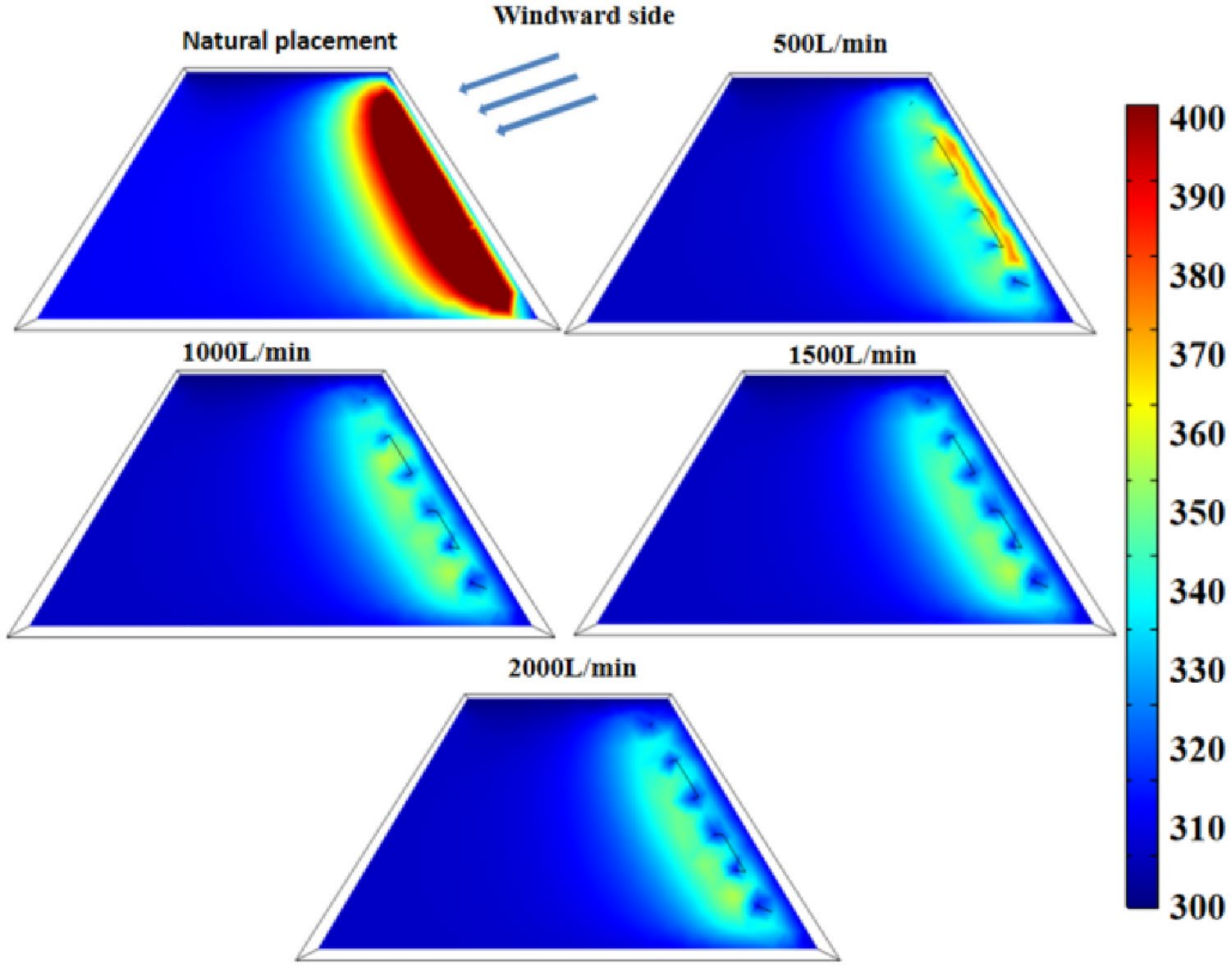
Temperature field on the 70th day before and after the arrangement of multi-row water-cooling steel pipes.

Figure 21 shows the temperature change at the temperature monitoring line ② on the 70th day with different flow rates in the pipe. Figure 22 shows the temperature field change before and after the arrangement of multi-row water-cooling steel pipes. As can be seen from the figure, the coal temperature of the coal pile is effectively controlled after the water-cooling steel pipe is arranged. When the flow rate is 500 L/min, there is still a small area of high temperature near the wind surface of the coal pile. When the flow rate is greater than 1000 L/min, the coal body temperature is controlled below the self-heating stage at 70 d. When the flow rate is 1500 L/min, the lowest coal temperature is reduced to 310 K, and the highest coal temperature is also below 343 K, which does not enter the self-heating stage. Compared with the multi-discharge water supply flow of 500 L/min, the highest coal temperature is reduced by 20 K. The temperature change at the flow rate of 2000L/min is basically the same as that at the flow rate of 1500L/min. Therefore, 1500 L/min is the optimal flow rate.

**Figure 23:**
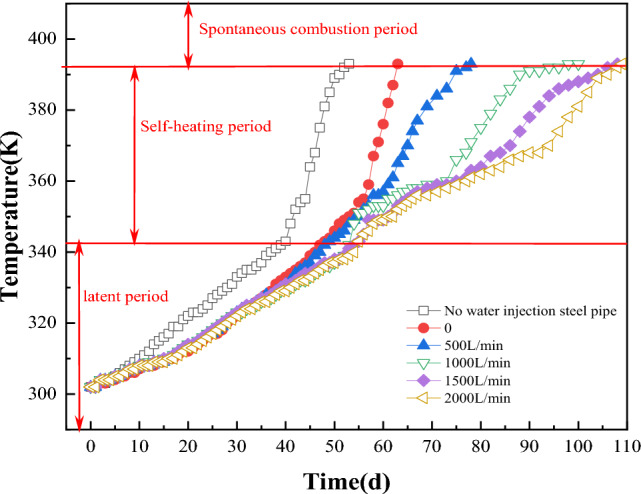
Temperature changes before and after arranging multiple rows of water-cooling steel pipes.

Figure 23 shows the change curve of coal temperature with time under different flow rates. As can be seen from Fig. 23, when no water-cooling steel pipe is arranged, the coal pile is placed for 40d naturally, and the maximum temperature reaches 343 K, entering the self-heating stage. At 53 d, the highest temperature reached 393 K and entered the spontaneous combustion stage.

The time for coal pile to enter the self-heating and self-combustion period is prolonged with the increase of the flow rate, and the results are shown in Table 6.

**Table 6:** Time for coal pile to enter self-heating and self-ignition period under different flow rates.

Flow L/min	Time to enter the self-heating period (d)	Time to enter the spontaneous combustion period (d)
0	48	63
500	51	78
1000	53	100
1500	55	108
2000	56	109

It can be seen that the time of coal pile entering self-heating period is extended by 8~19 d and the time of coal pile entering self-combustion period is extended by 10~56 d after the arrangement of water-cooling steel pipe.

## Conclusion

This paper uses COMSOL numerical simulation software to analyze the spontaneous ignition process and the prevention effects on open pit coal piles. Based on the law of natural ignition of coal piles and the range of the high-temperature area, this paper proposes a scheme to cool coal piles using water-cooling steel pipes, it studies the effect of water-cooling steel pipes on the temperature field distribution in open pit coal piles, and provides the optimal layout parameters for water-cooling steel pipes in coal piles. The main conclusions are as follows:

1. The cooling radius of the water-cooling steel pipe is determined by the heat transfer coefficient of water, the heat value of coal, and the outer diameter of the steel pipe. However, it is independent of the flow rate and water temperature. Under simulated conditions, the cooling radius of the water-cooling steel pipe is 0.5 m. The optimal cooling effect can be achieved when the center distance between adjacent steel pipes is 1 m.
2. The water flow in the water-cooling pipe greatly influences the cooling effect of the coal pile. The higher the flow rate, the greater the cooling range of the coal pile. For a single pipe arrangement, the optimal flow rate is 500L/min, while for a multi-row arrangement, it is 1500L/min.
3. The cooling effect of the coal pile is negatively correlated with the water inlet temperature. The lower the water temperature at the inlet of the water-cooling pipe, the better the cooling effect of the coal pile.
4. The time for the coal pile to enter the self-heating period is extended by 8 to 19 days, and the time for the coal pile to enter the self-combustion period is extended by 10 to 53 days after the arrangement of multi-row water-cooling steel pipes.

## Data Availability

The datasets used and/or analyzed during the current study are available from the corresponding author on reasonable request.
